# A yeast metabolome-based model for an ecotoxicological approach in the management of lignocellulosic ethanol stillage

**DOI:** 10.1098/rsos.180718

**Published:** 2019-01-16

**Authors:** Luca Roscini, Lorenzo Favaro, Laura Corte, Lorenzo Cagnin, Claudia Colabella, Marina Basaglia, Gianluigi Cardinali, Sergio Casella

**Affiliations:** 1 Department of Pharmaceutical Sciences-Microbiology, CEMIN, Centre of Excellence on Nanostructured Innovative Materials, University of Perugia, Perugia, Italy; 2 Department of Chemistry, Biology and Biotechnology, CEMIN, Centre of Excellence on Nanostructured Innovative Materials, University of Perugia, Perugia, Italy; 3 Department of Agronomy Food Natural resources Animals and Environment (DAFNAE), University of Padova, Legnaro, Italy

**Keywords:** FTIR, lignocellulosic stillage, modelling, *Saccharomyces cerevisiae*, stress response

## Abstract

Lignocellulosic bioethanol production results in huge amounts of stillage, a potentially polluting by-product. Stillage, rich in heavy metals and, mainly, inhibitors, requires specific toxicity studies to be adequately managed. To this purpose, we applied an FTIR ecotoxicological bioassay to evaluate the toxicity of lignocellulosic stillage. Two weak acids and furans, most frequently found in lignocellulosic stillage, have been tested in different mixtures against three *Saccharomyces cerevisiae* strains. The metabolomic reaction of the test microbes and the mortality induced at various levels of inhibitor concentration showed that the strains are representative of three different types of response. Furthermore, the relationship between concentrations and FTIR synthetic stress indexes has been studied, with the aim of defining a model able to predict the concentrations of inhibitors in stillage, resulting in an optimized predictive model for all the strains. This approach represents a promising tool to support the ecotoxicological management of lignocellulosic stillage.

## 1. Introduction

Stillage is the liquid by-product obtained after the distillation of ethanol following fermentation. The production of bioethanol from any biological substrate results in high volumes of stillage which displays a great pollution potential. For each litre of ethanol manufactured, about 20 l of stillage can be produced, with a chemical oxygen demand (COD) of about 100 g l^−1^ [1–3]. A medium-sized ethanol plant, making a million litres of fuel per year, generates stillage with a pollution level equivalent to the sewage of a small city [4]. Stillage obtained from the distillation of fermented mash is quite hot (70–80°C), deep brown and has high levels of organic materials and solids. Nevertheless, the pollution potential of the effluent depends on feedstocks, facility and methods applied to recover the alcohols [1,3]. Additional environmental concerns on stillage would be provided by high N or sulfate content, colour, heavy metal concentrations and the presence of organic pollutants [1,3].

Whereas the industrial-scale conversion of sugars and starchy crops into ethanol is a mature technology [5–8], large-scale production from lignocellulosic biomass has limited applications. Nevertheless, efforts are ongoing to improve economics and to move cellulose-to-biofuels conversion into production [9–13]. In contrast to sugar- and starch-rich materials, the great availability of lignocellulosic biomass means that ethanol processing from lignocellulosic biomass could replace a major portion of fossil fuels [11,12]. However, due considerations for treatment and utilization of the stillage are essential to qualify lignocellulosic ethanol as a sustainable ‘green energy’ route [14].

Commonly, the traits of stillage from cellulosic materials are comparable to those of conventional substrates (i.e. sugarcane or corn) and, therefore, the ways so far developed to treat and use stillage from conventional feedstocks could also be valid for lignocellulosic materials.

However, the higher levels of heavy metals together with the occurrence of unusual inhibitory compounds associated with the feedstock pre-treatment process, such as weak acids, furans and phenolic molecules [12,15–17], call for ecotoxicological studies to carefully assess their toxic effects before being treated and/or disposed.

**Figure 1.**
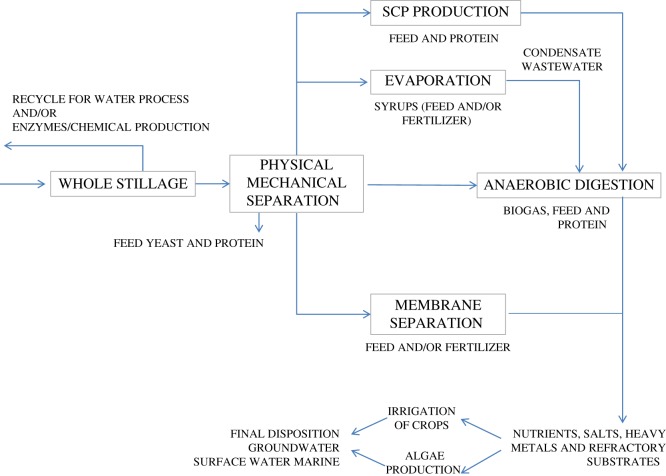
Stillage treatment technology and utilization options (modified and expanded from [3]). SCP stands for single cell protein.

Figure 1 exemplifies the main stillage treatment and valorization technologies. In order to recover and remove suspended solids containing yeast and other materials, physical/mechanical separations can be implemented. In the case of a corn-to-ethanol process, the separated solids can be dried and sold as a high-value animal feed called dried distillers grains (DDGs) [6,18].

After mechanical treatment, a cluster of technologies is available including single cell protein, membrane separation, evaporation, enzyme(s) production and anaerobic digestion (figure 1) [1,3,4,15,19–21]. In the case of lignocellulosic stillage, occurrence and levels of inhibitory compounds will greatly influence stillage valorization technologies [1,22]. In specific circumstances, proper dilution rates and/or flow of different valorization strategies will be useful to reduce the inhibitor content to safety levels, in order to convert stillage into valuable products. Thus, towards the widespread implementation of lignocellulosic ethanol plants, safe stillage management procedures will be of great impact and new ecotoxicological insights are needed.

Only a few ecotoxicological studies have taken into consideration the problem related to the toxicity of the disposed stillages, most of them focused on the genotoxicity assessment on higher eukaryotes [23–25].

In this perspective, this study proposes a yeast metabolome-based assay to evaluate the levels of lignocellulosic inhibitors commonly found in stillage. Lower eukaryotes present a number of advantages such as easy manipulation and possibility of testing simultaneously several different experimental conditions on millions of cells in different cell cycle phases. For this purpose, three *Saccharomyces cerevisiae* strains, with different tolerance to inhibitors, have been chosen as biological sensors, with the possibility to extend the results of this bioassay also to higher eukaryotes [26]. Fourier transform infrared (FTIR) spectroscopy was employed to detect the reaction of test organisms to different mixtures of inhibitors as a paradigm of different lignocellulosic stillage compositions [9,11,12,27,28]. This procedure has already been successfully applied for the assessment of stress-induced cell status in response to various stressors [29,30]. Finally, an appropriate modelling has been developed in order to predict the concentration of these compounds in stillages. This ecotoxicological approach, coupling the above-mentioned advantages related to the use of microorganisms as biological sensors to the high efficiency and low cost of FTIR, is proposed as a tool for the correct management of this environmentally unfriendly waste.

## 2. Material and methods

### 2.1. Cultures and growth conditions

FTIR-based bioassay was performed with three *S. cerevisiae* strains, Fp84, Fm17 and DSM70449, selected among 160 yeast strains already described for their tolerance to lignocellulosic inhibitory compounds [17,27]. Distribution analysis of the inhibitors tolerance was obtained by using a box-and-whiskers plot.

For long-term storage, yeast strains were conserved at −80°C on 15% (v/v) glycerol and 85% (v/v) YPD broth (yeast extract 1%, peptone 1% and dextrose 2%, Difco Laboratories, USA). Pre-cultures were inoculated at optical density (OD_600_) = 0.2 in 500 ml bottles containing 50 ml YPD medium and grown for 18 h at 25°C, with 150 r.p.m. shaking.

### 2.2. Stressing agents

Weak acids and furans obtained from Sigma (www.sigmaaldrich.com) were used at increasing levels in distilled sterile water. Each dose of inhibitory compound was reported as three relative concentrations (RCs, low, medium and high) according to the low, medium and high inhibitor levels usually found in lignocellulosic stillage [12,17,27,28]. The low, medium and high RCs consisted of (mM): 30, 60 and 120 for acetic acid; 13, 27 and 53 for formic acid; 7, 14 and 29 for furfural; and 7, 15 and 30 for 5-(hydroxymethyl)furfural (HMF), respectively.

Inhibitors were also formulated into binary, ternary and quaternary mixtures obtained with increasing concentrations of weak acids or furans. pH values of each solution of single acid ranged from 2.3 to 2.7. In the case of furan preparations, pH values were about 6.5. pH values of the binary and ternary mixtures ranged between 2.3 and 2.7; meanwhile quaternary inhibitors mixtures had the pH values of 2.6, 2.5, 2.4, 2.2 for RC_L_, RC_M_ and RC_H_.

### 2.3. Ecotoxicological analysis

An FTIR bioassay was used to assess the toxicity of inhibitory compounds both as single and as binary, ternary and quaternary mixtures. Each cell suspension was centrifuged (3 min at 5300*g*), washed twice with distilled sterile water and re-suspended with proper volumes of distilled water to obtain a final concentration of 2.5 × 10^8^ cell ml^−1^. Inhibitors were added in order to obtain the RCs reported in the Stressing agents section. The control (RC_0_, no inhibitors) was obtained by re-suspending cells in distilled sterile water. All tests were performed in triplicate. Tubes were incubated for 1 h at 25°C in a shaking incubator set at 50 r.p.m. After the incubation, cells were prepared for FTIR experiments as described by Corte and colleagues [31]. The biocidal activity tests were carried out in parallel to compare the metabolomic changes with the loss of viability induced by inhibitors. A 100 µl volume of each cell suspension prepared for the FTIR analysis was serially diluted to determine the viable cell counting, in triplicate, on YPDA with chloramphenicol (0.5 g l^−1^). The biocidal effect of the tested compounds was highlighted as cell mortality induced at different concentrations. The cell mortality (*M*) was calculated as *M* = (1 − Cv/Ct) × 100, where Cv is the number of viable cells in the tested sample and Ct the number of viable cells in the control suspension.

### 2.4. FTIR analysis

Each culture sample was analysed in triplicate. For each strain, 105 µl volume was sampled for three independent FTIR readings (35 µl each, according to the technique suggested by Manfait and colleagues [30]). FTIR measurements were performed in transmission mode. All spectra were recorded in the range between 4000 and 400 cm^−1^ with a TENSOR 27 FTIR spectrometer, equipped with HTS-XT accessory for rapid automation of the analysis (BRUKER Optics GmbH, Ettlingen, Germany). Spectral resolution was set at 4 cm^−1^, sampling 256 scans per sample to obtain high quality spectra (signal to noise ratio values greater than 4000 within the 2100–1900 cm^−1^ interval). The software OPUS version 6.5 (BRUKER Optics GmbH, Ettlingen, Germany) was used to assess the quality test, subtract the interference of atmospheric CO_2_ and water vapour, correct baseline (rubberband method with 64 points) and to apply vector normalization to the whole spectra. Spectral data matrices were exported as ASCII text from Opus 6.5 and analysed using the ‘R’ script MSA (metabolomic spectral analysis) according to Cardinali and colleagues [32]. Six synthetic stress indexes (SI) were calculated as normalizations of the Euclidean distances between the response spectra of cells under stress and those of cells maintained in water, according to the procedure proposed by Corte and co-workers [29]. Briefly, Euclidean distances among different conditions were calculated using the vectors of the corresponding intensities. Since the five selected spectral regions have different lengths, the normalization was carried out by dividing the Euclidean distance value by the ratio between the number of points of the whole spectrum and the number of points of each specific spectral region. One SI is related to the entire spectrum (global stress index, GSI) while the other five are related to the five different spectral regions defined as follows: fatty acids (W1) from 3000 to 2800 cm^−1^, amides (W2) from 1800 to 1500 cm^−1^, mixed region (W3) from 1500 to 1200 cm^−1^, carbohydrates (W4) from 1200 to 900 cm^−1^ and typing region (W5) from 900 to 700 cm^−1^ [30]. The typing region was not considered in this analysis because its response did not appear correlated with the specific stressing conditions tested.

### 2.5. Modelling and statistical analyses

Regression analysis, modelling and statistics were performed using mathematical and statistical functions under MS Excel and ‘R’ (www.cran.org), respectively. R base package and ‘R’ script MSA [32] has been used to calculate SI. According to the distribution of results, parametric and/or non-parametric statistics were applied in MS Excel to test hypotheses on the means and/or the medians, respectively. The *α* value was set to 0.05 and the level of significant difference to *p* < 0.05. Pearson linear regression analysis was applied to test the best fitting between RC and FTIR data (MS Excel). Statistical significance of the general models was assessed by Student *t*-Test (MS Excel).

## 3. Results and discussion

### 3.1. Inhibitor tolerance assessed in robust yeast strains

**Figure 2.**
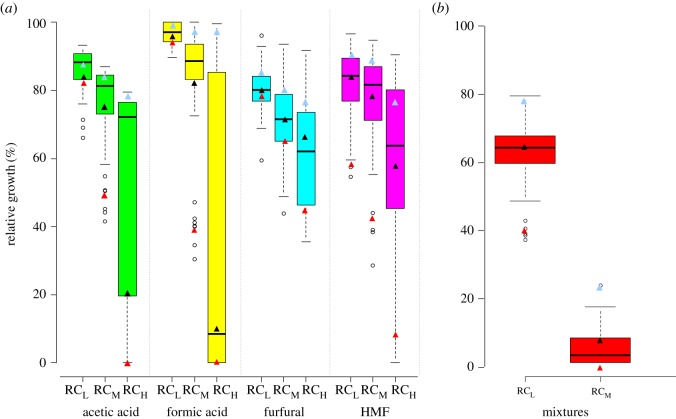
Box-and-whiskers plot distribution analysis of inhibitor tolerance of 160 *S. cerevisiae* strains to inhibitors, alone and in mixtures. (*a*) Distribution analysis of inhibitor tolerance to single inhibitors, each tested at three different concentrations; (*b*) distribution analysis of inhibitor tolerance to inhibitor mixtures at two concentrations (RC_L_ and RC_M_) obtained combining the first two concentrations of each inhibitor. Values are reported as relative growth (%) by assessing the growth after 48 h in YPD medium with and without the inhibitory compounds. Triangles represent the position of *S. cerevisiae* Fm17 (light blue), Fp84 (black) and DSM70449 (red).

Three *S. cerevisiae* yeast, namely Fm17, Fp84 and DSM70449, were chosen as test organism among 160 strains previously assessed for inhibitor tolerance when grown in complex or minimal broths with increasing concentrations of inhibitory compounds both as single and as quaternary mixtures. Inhibitor tolerance was calculated as relative growth (OD value, %) by assessing the growth in the medium with and without the inhibitory compounds (acetic acid, formic acid, furfural and HMF). Three increasing RC (low, medium and high) have been tested (figure 2) and the position of the three strains evaluated in this study (Fm17, Fp84 and DSM70449) is reported for the tested inhibitor concentrations.

The distribution analysis of single inhibitor tolerance showed a great variability with ranges becoming larger at higher inhibitor concentrations, mainly for the acetic and formic acid (figure 2*a*). Quaternary mixtures caused a growth decrease proportional to their concentrations without variability range increase (figure 2*b*) otherwise observed after single inhibitor exposure (figure 2*a*). Moreover, the highest relative concentration of the quaternary mixtures was lethal for all the strains (data not shown).

**Figure 3.**
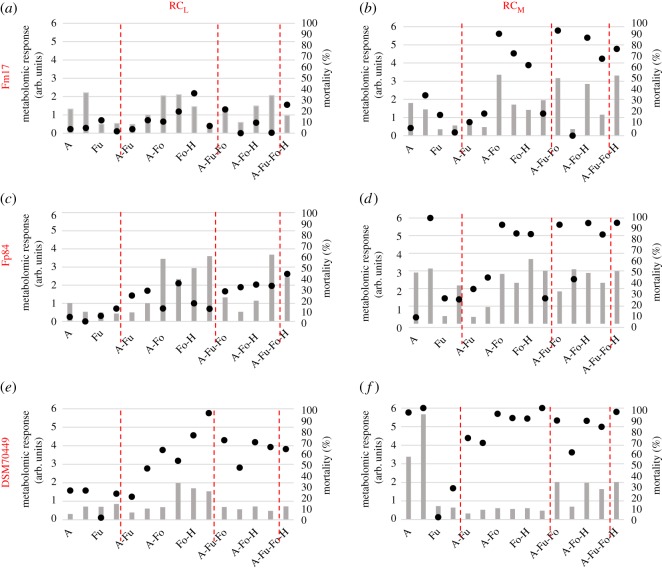
Stress response (GSI) and mortality of Fm17, Fp84 and DSM70449 strains challenged by single inhibitors, binary, ternary or quaternary inhibitor mixtures at low and medium relative concentrations (RC_L_ and RC_M_). Grey bars represent GSI calculated as normalizations of the Euclidean distances between the response spectra of cells under stress and those of cells maintained in water. The degree of variability between replicas throughout the FTIR spectra ranged around 2.5 × 10^−2^. Black dots represent mortality values as the mean of three replicates with the relative standard error always being less than 5% (not reported).

Overall, *S. cerevisiae* Fm17 is always positioned at the top of single and combined distributions, whereas DSM70449 was placed at the bottom and Fp84 close to the average. The three strains were previously characterized on the basis of their stress response to these four inhibitors exhibiting specific metabolomic reactions in agreement to their tolerance levels [33] and could be useful as biological sensors of the stress induced by lignocellulosic inhibitors. In order to define whether the conclusions reached by Favaro and colleagues [33] are representative of all the combinations of inhibitors potentially present in lignocellulosic stillage, we tested the stressing effect exerted by single compounds, binary, ternary and quaternary mixtures on these test organisms. To this purpose, each strain has been exposed to three different concentrations (RC_L_, RC_M_ and RC_H_) and evaluated in terms of mortality and metabolomic response synthesized as GSI. As RC_H_ was lethal in all the combinations, only data related to RC_L_ and RC_M_ are reported in figure 3.

Each strain exhibited a specific pattern of mortality. Specifically, acetic acid and furans induced the lowest biocidal effect whereas high mortality was observed in most of the combinations containing formic acid.

In general, the higher mixtures concentration induced the higher mortality levels in all tested microorganisms. *S. cerevisiae* DSM70449 was confirmed to be the most sensitive strain (figure 3*e,f*), while the Fp84 and the Fm17 showed intermediate and high inhibitor tolerance, respectively (figure 3*a*–*d*).

The picture depicted by coupling the metabolomic reaction (GSI) with the mortality induced by the inhibitory compounds at concentration non-saturating mortality (RC_L_) confirmed that *S. cerevisiae* Fp84, Fm17 and DSM70449 are indeed representative of three different types of response to the inhibitory compounds most commonly present in bioethanol plants.

Specifically, *S. cerevisiae* Fm17 presents the traits characteristic of a resistant strain, in which a low mortality is accompanied by a low metabolomic reaction. Vice versa, the strain DSM70449 displays the highest mortality and the lowest GSI values, typical of a sensitive organism which is not able to react to the strong and immediate action exerted by a toxic compound [34]. Finally, *S. cerevisiae* Fp84 shows low mortality values together with a relatively high metabolomic response, indicating an intermediate tolerance to these stressing agents.

### 3.2. Modelling

#### 3.2.1. Primary models

The outcome of the mortality and GSI, determined with the FTIR bioassay, confirmed that the three strains, chosen on the basis of relative growth rates, are also representative of three different styles of response to all the combinations of the stressing agents tested. This piece of evidence led us to concentrate exclusively on the response to the quaternary cocktail, as the most complex stressing condition and one of the more frequently encountered in bioethanol plants and related stillage by-products. In order to define a model to predict the concentrations of inhibitors on the basis of the FTIR spectrum of cells under stress, we studied the relationship between RC and the SIs in different spectral regions or the spectrum as a whole (GSI). This operation was carried out for the three strains separately. The regression analyses showed that a second degree correlation curve described this relationship quite well (*R*^2^ 0.99, 0.96 and 0.83 for Fp84, DSM70449 and Fm17, respectively). According to these analyses, we formulated first independent primary models for W1, W2, W3, W4 and the whole spectrum, as reported in

3.1 $${\mathrm{RC}}_{\mathrm{sw}}=a{\mathrm{SI}}_{\mathrm{sw}}^{2}+b{\mathrm{SI}}_{\mathrm{sw}}+c_{\mathrm{sw},}$$

where RC_sw_ indicates the relative concentration estimated on the basis of the response of a specific spectral area (w) in a specific strain acting as a biosensor (s) while SI stands for the stressing index of the wth spectral region considered. The parameters obtained for the 15 equations (3 strains analysed in 5 regions) based on the experimental data are listed in table 1.

**Table 1.** Parameters obtained for the construction of the primary model equations. W1, W2, W3 and W4 represent, respectively, the fatty acids region (3000–2800 cm^−1^), the amides region (1800–1500 cm^−1^), the mixed region from 1500 to 1200 cm^−1^ and the carbohydrates one from 1200 to 900 cm^−1^. WS stands for whole spectrum.

		parameters			
strain	spectral region	*a*	*b*	*c*	*R*^2^ value
Fp84	W1	0.997	13.056	0.142	0.498
	W2	0.895	1.553	0.078	0.938
	W3	2.544	−10.997	−0.134	0.990
	W4	−0.989	15.862	−1.344	0.502
	WS	43.077	−96.031	0.063	0.990
DSM70449	W1	3.198	9.209	7.156	0.960
	W2	0.679	1.147	5.264	0.906
	W3	1.592	2.410	7.418	0.941
	W4	14.301	−17.229	−0.122	0.998
	WS	9.412	8.529	4.426	0.957
Fm17	W1	2.093	3.819	5.399	0.950
	W2	−0.005	5.776	5.083	0.785
	W3	−2.405	31.267	−9.201	0.846
	W4	−2.658	25.816	−1.647	0.503
	WS	2.990	10.959	3.898	0.824

#### 3.2.2. General model

After defining the primary models, a general model was determined for each strain by assigning a specific weight to each primary model equation, and therefore to the contribution of each spectral region, as reported in

3.2 $$\begin{array}{rl} \;RC_{s}= & \frac{\sum_{i=1}^{i=4}w_{i}(a{\mathrm{SI}}_{i}^{2}+bSI_{i}+c_{i})}{\sum_{i=1}^{i=4}w_{i}}\\ & =\frac{\begin{array}{c} w1(a{\mathrm{SI}}_{W1}^{2}+bSI_{W1}+c_{W1})+w2(a{\mathrm{SI}}_{W2}^{2}+bSI_{W2}+c_{W2})+w3(a{\mathrm{SI}}_{W3}^{2}+bSI_{W3}+c_{W3})\\ +w4(a{\mathrm{SI}}_{W4}^{2}+bSI_{W4}+c_{W4})+\mathrm{wS}(a{\mathrm{SI}}_{\mathrm{WS}}^{2}+bSI_{\mathrm{WS}}+c_{\mathrm{WS}}) \end{array}}{w1+w2+w3+w4+\mathrm{wS}}, \end{array}$$

where RC_s_ indicates the relative concentration estimated by each biosensor, while w1, w2, w3, w4 and wS are the specific weights assigned to each primary model. Two different models were evaluated, the first based on the response of the entire spectrum (wS) while the second on that of W1, W2, W3 and W4 regions, separately.

##### 3.2.2.1. General model based on the response of the whole FTIR spectrum of cells under stress

RC_s_ has been estimated by assigning an entire weight to the primary model of the whole spectrum (wS = 1) and nullifying the contribution of all other equations (w1, w2, w3 and w4 = 0), as reported in table 2.

**Table 2.** Relative concentration predicted for each biosensor of low, medium and high RCs of quaternary inhibitory mixtures. Upper and lower sections report data obtained by assigning an entire weight to the primary model of the whole spectrum or a specific weight to each primary model equation for W1, W2, W3 and W4 regions, respectively. w1, w2, w3, w4 and wS represent the specific weights assigned to the primary models equations W1, W2, W3, W4 and whole spectrum, respectively.

	specific weight					observed RCs					quartiles		
biosensor	_w1_	_w2_	_w3_	_w4_	_wS_	RC_L_	RC_M_	RC_H_	corr	*T*-test	RC_L_	RC_M_	RC_H_
Fp84	0	0	0	0	1	21.61	56.09	97.24	0.99	1.00	1	2	4
DSM70449	0	0	0	0	1	15.54	60.00	95.01	0.97	0.97	1	2	4
Fm17	0	0	0	0	1	17.40	72.86	80.84	0.83	0.97	1	3	4
expected RCs						25.00	50.00	100.00			1	2	4
Fp84	0	0	1	0	0	29.48	44.07	100.00	0.99	0.99	1	2	4
DSM70449	0.1	0	0.1	0.8	0	24.30	49.59	99.75	1.00	0.99	1	2	4
Fm17	1	0	0	0	0	14.55	60.33	94.72	0.96	0.96	1	2	4
expected RCs						25.00	50.00	100.00			1	2	4

Predicted values were also classified as classified relative concentration (CRC) according to the equation (3.3) that rounds to the nearest integer in a scale 1–2–4, corresponding to the quartiles of classification of the three RCs tested (RC_L_, RC_M_, RC_H_).

3.3 $$\mathrm{CRC}=\frac{\mathrm{RC}}{25}+\;\frac{1}{2}$$

This model proved to be effective in the prediction of RCs only using the Fp84 and DSM70449 as sensors. In fact, *t*-test analysis showed no significant differences between the observed and the expected data (Fp84, *p*-value = 1; DSM70449, *p*-value = 0.97). Furthermore, the observed and expected RCs classification matched perfectly (table 2). On the contrary, the primary model based on the response of the whole FTIR spectrum does not work equally well with the test strain Fm17. The correlation between the two datasets was slightly lower (0.83) and the classification of predicted RCs corresponded to that of the observed values only for the RC_H_ and RC_L_. The intermediate concentration (RC_M_) resulted overestimated, falling in the third quartile rather than in the second.

##### 3.2.2.2. General model based on the response of the different FTIR spectral regions

As reported in table 2, RC_s_ has also been estimated by assigning a specific weight to each primary model equation for W1, W2, W3 and W4 regions, according to the *R*^2^ values reported in table 1. In this second modelling, the contribution of the whole spectrum was nullified (wS = 0).

The use of the weighted equations from the four spectral regions (W1–W4) optimized the model predictivity for all strains and ranges of concentrations tested (*p*-values = 0.99, 0.99 and 0.96 for Fp84, DSM70449 and Fm17, respectively). Moreover, observed values resulted always classified in the same categories of the RCs expected data.

The best RCs prediction was obtained for the Fp84 strain on the basis of the sole response of the mixed region (w3 = 1) as well as for the resistant strain Fm17 on that of the fatty acids (w1 = 1). Conversely, the more predictive primary model for DSM70449 strain resulted that based on carbohydrates (w4 = 0.8), with a small contribution due to fatty acids and mixed regions models (w1 and w3 = 0.1).

The increased predictivity of the second model depends on the option to attribute a specific weight to each primary model equation considering only those areas which best respond to these specific stressing conditions. Using this procedure, regardless of the strain tolerance level, all test microorganisms become excellent sensors for the stressing conditions tested and, therefore, for the prediction of the RCs of inhibitory compounds normally found into lignocellulosic stillage.

Overall, the present study proposes a novel tool to plan proper dilution rates and/or flows for the ecotoxicological management of lignocellulosic stillages. The desirable diffusion of large-scale ethanol plants will require new simple and cheap approaches to reduce the inhibitor content to safe levels in view of different possible uses of stillage [1,2,12].

Furthermore, the final disposition of treated stillages will need rapid and reliable analysis of their inhibitor content to avoid any environmental hazard [14]. Moreover, most of the literature data refer to bench-scale systems and many post-disposal monitoring programmes are conducted in the short term, highlighting a consistent lack of data on the proper management of these waste and their reuse in the long term [35].

Furthermore, most of the bioassays so far reported are not tailored to quantify and classify the changes detected, without analysing the type of action and the strength of their activity on living cells. The approach applied in this work has already been designed to serve as an ecotoxicological assay and successfully applied in different fields including the qualitative and quantitative evaluation of the antagonistic effects of inhibitors mixtures on *S. cerevisiae* metabolism [33].

## 4. Conclusion

The present study provides a novel approach to support the ecotoxicological management of lignocellulosic stillage based on the modelling of FTIR spectra of *S. cerevisiae* cells challenged with quaternary cocktails of lignocellulosic inhibitors.

Compared to the traditional chemical analyses, FTIR spectroscopy offers the advantage of a rapid, non-invasive and cheap method also exploitable with a handheld instrument. This would open the possibility of testing the actual effect of the stillage, or any other type of compound or complex mixtures of toxic compounds, on living cells, and would be very useful in environmental sciences.

All test microorganisms resulted in excellent sensors for the stressing conditions tested and, therefore, for the prediction of the RCs of inhibitory compounds normally found in lignocellulosic stillage. The choice of *S. cerevisiae* cells as biosensors represents a valid model because their eukaryotic nature could allow the extension of the results of this bioassay also to other eukaryotes, including plants and man, while their microbial nature is ideal for the laboratory usage. Furthermore, depending on the case, the procedure could easily be applied to other test organisms through a simple *a priori* calibration of the model.

Finally, this bioassay can be further upgraded by producing large FTIR libraries with the actual stillages coming from productive plants, improving the level of predictability without limiting the ease of application. The application of artificial neural networks (ANN) is a further perspective to potentiate this bioassay.

## Supplementary Material

Supplementary Figures

## Supplementary Material

Titles and captions of ESM

## Data accessibility

All the performance data are provided in the main text. FTIR spectra are available as electronic supplementary material.

## References

[1] Mohana S, Acharya BK, Madamwar D 2009 Distillery spent wash: treatment technologies and potential applications. J. Hazard. Mater. 163, 12–25. (10.1016/j.jhazmat.2008.06.079) 18675513

[2] Oosterkamp MJ, Méndez-García C, Kim C-H, Bauer S, Ibáñez AB, Zimmerman S, Hong P-Y, Cann IK, Mackie RI 2016 Lignocellulose-derived thin stillage composition and efficient biological treatment with a high-rate hybrid anaerobic bioreactor system. Biotechnol. Biofuels 9, 120 (10.1186/s13068-016-0532-z) 27274357 PMC4895995

[3] Wilkie AC, Riedesel KJ, Owens JM 2000 Stillage characterization and anaerobic treatment of ethanol stillage from conventional and cellulosic feedstocks. Biomass Bioenergy 19, 63–102. (10.1016/S0961-9534(00)00017-9)

[4] Mirsepasi A, Honary H, Mesdaghinia A, Mahvi A, Vahid H, Karyab H 2006 Performance evaluation of full scale UASB reactor in treating stillage wastewater. J. Environ. Health Sci. Eng. 3, 79–84.

[5] Favaro L, Viktor MJ, Rose SH, Viljoen-Bloom M, van Zyl WH, Basaglia M, Cagnin L, Casella S 2015 Consolidated bioprocessing of starchy substrates into ethanol by industrial *Saccharomyces cerevisiae* strains secreting fungal amylases. Biotechnol. Bioeng. 112, 1751–1760. (10.1002/bit.25591) 25786804

[6] Bothast R, Schlicher M 2005 Biotechnological processes for conversion of corn into ethanol. Appl. Microbiol. Biotechnol. 67, 19–25. (10.1007/s00253-004-1819-8) 15599517

[7] Favaro L, Basaglia M, Saayman M, Rose S, van Zyl WH, Casella S 2010 Engineering amylolytic yeasts for industrial bioethanol production. Chem. Eng. Trans. 20, 97–102.

[8] Favaro L, Jooste T, Basaglia M, Rose SH, Saayman M, Görgens JF, van Zyl WH, Casella S 2013 Designing industrial yeasts for the consolidated bioprocessing of starchy biomass to ethanol. Bioengineered 4, 97–102. (10.4161/bioe.22268) 22989992 PMC3609629

[9] Balat M, Balat H 2009 Recent trends in global production and utilization of bio-ethanol fuel. Appl. Energy 86, 2273–2282. (10.1016/j.apenergy.2009.03.015)

[10] Cripwell R, Favaro L, Rose SH, Basaglia M, Cagnin L, Casella S, van Zyl W 2015 Utilisation of wheat bran as a substrate for bioethanol production using recombinant cellulases and amylolytic yeast. Appl. Energy 160, 610–617. (10.1016/j.apenergy.2015.09.062)

[11] Kumar R, Singh S, Singh OV 2008 Bioconversion of lignocellulosic biomass: biochemical and molecular perspectives. J. Ind. Microbiol. Biotechnol. 35, 377–391. (10.1007/s10295-008-0327-8) 18338189

[12] Limayem A, Ricke SC 2012 Lignocellulosic biomass for bioethanol production: current perspectives, potential issues and future prospects. Prog. Energy Combust. Sci. 38, 449–467. (10.1016/j.pecs.2012.03.002)

[13] Treusch K, Ritzberger J, Schwaiger N, Pucher P, Siebenhofer M 2017 Diesel production from lignocellulosic feed: the bioCRACK process. R. Soc. open sci. 4, 171122 (10.1098/rsos.171122) 29291098 PMC5717672

[14] Falano T, Jeswani HK, Azapagic A 2014 Assessing the environmental sustainability of ethanol from integrated biorefineries. Biotechnol. J. 9, 753–765. (10.1002/biot.201300246) 24478110 PMC4674963

[15] Barta Z, Reczey K, Zacchi G 2010 Techno-economic evaluation of stillage treatment with anaerobic digestion in a softwood-to-ethanol process. Biotechnol. Biofuels 3, 21 (10.1186/1754-6834-3-21) 20843330 PMC2945328

[16] Lu Y, Cheng Y-F, He X-P, Guo X-N, Zhang B-R 2012 Improvement of robustness and ethanol production of ethanologenic *Saccharomyces cerevisiae* under co-stress of heat and inhibitors. J. Ind. Microbiol. Biotechnol. 39, 73–80. (10.1007/s10295-011-1001-0) 21698486

[17] Favaro L, Basaglia M, Casella S 2014 Innately robust yeast strains isolated from grape marc have a great potential for lignocellulosic ethanol production. Ann. Microbiol. 64, 1807–1818. (10.1007/s13213-014-0826-y)

[18] Favaro L, Cagnin L, Basaglia M, Pizzocchero V, van Zyl WH, Casella S 2017 Production of bioethanol from multiple waste streams of rice milling. Bioresour. Technol. 244, 151–159. (10.1016/j.biortech.2017.07.108) 28779666

[19] Cavka A, Alriksson B, Rose SH, van Zyl WH, Jönsson LJ 2014 Production of cellulosic ethanol and enzyme from waste fiber sludge using SSF, recycling of hydrolytic enzymes and yeast, and recombinant cellulase-producing *Aspergillus niger*. J. Ind. Microbiol. Biotechnol. 41, 1191–1200. (10.1007/s10295-014-1457-9) 24862324

[20] Skovgaard PA, Christensen BH, Felby C, Jørgensen H 2014 Recovery of cellulase activity after ethanol stripping in a novel pilot-scale unit. J. Ind. Microbiol. Biotechnol. 41, 637–646. (10.1007/s10295-014-1413-8) 24549412

[21] Zhang CM, Mao ZG, Wang X, Zhang JH, Sun FB, Tang L, Zhang HJ 2010 Effective ethanol production by reutilizing waste distillage anaerobic digestion effluent in an integrated fermentation process coupled with both ethanol and methane fermentations. Bioprocess. Biosyst. Eng. 33, 1067–1075. (10.1007/s00449-010-0432-8) 20473528

[22] Monlau F, Sambusiti C, Barakat A, Quéméneur M, Trably E, Steyer J-P, Carrère H 2014 Do furanic and phenolic compounds of lignocellulosic and algae biomass hydrolyzate inhibit anaerobic mixed cultures? A comprehensive review. Biotechnol. Adv. 32, 934–951. (10.1016/j.biotechadv.2014.04.007) 24780154

[23] Correia J, Christofoletti C, Marcato A, Marinho J, Fontanetti C 2017 Histopathological analysis of tilapia gills (*Oreochromis niloticus* Linnaeus, 1758) exposed to sugarcane vinasse. Ecotoxicol. Environ. Saf. 135, 319–326. (10.1016/j.ecoenv.2016.10.004) 27770647

[24] Correia JE, Christofoletti CA, Ansoar-Rodríguez Y, Guedes TA, Fontanetti CS 2017 Comet assay and micronucleus tests on *Oreochromis niloticus* (Perciforme: Cichlidae) exposed to raw sugarcane vinasse and to physicochemical treated vinasse by pH adjustment with lime (CaO). Chemosphere 173, 494–501. (10.1016/j.chemosphere.2017.01.025) 28131919

[25] Garcia CFH, de Souza RB, de Souza CP, Christofoletti CA, Fontanetti CS 2017 Toxicity of two effluents from agricultural activity: comparing the genotoxicity of sugar cane and orange vinasse. Ecotoxicol Environ. Saf. 142, 216–221. (10.1016/j.ecoenv.2017.03.053) 28412625

[26] Corte L, Roscini L, Zadra C, Antonielli L, Tancini B, Magini A, Emiliani C, Cardinali G 2012 Effect of pH on potassium metabisulphite biocidic activity against yeast and human cell cultures. Food Chem. 134, 1327–1336. (10.1016/j.foodchem.2012.03.025) 25005950

[27] Favaro L, Basaglia M, Trento A, Van Rensburg E, García-Aparicio M, Van Zyl WH, Casella S 2013 Exploring grape marc as trove for new thermotolerant and inhibitor-tolerant *Saccharomyces cerevisiae* strains for second-generation bioethanol production. Biotechnol. Biofuels 6, 1 (10.1186/1754-6834-6-168) 24286305 PMC4176503

[28] Martín C, Jönsson LJ 2003 Comparison of the resistance of industrial and laboratory strains of *Saccharomyces* and *Zygosaccharomyces* to lignocellulose-derived fermentation inhibitors. Enzyme Microb. Technol. 32, 386–395. (10.1016/S0141-0229(02)00310-1)

[29] Corte L, Rellini P, Roscini L, Fatichenti F, Cardinali G 2010 Development of a novel, FTIR (Fourier transform infrared spectroscopy) based, yeast bioassay for toxicity testing and stress response study. Anal. Chim. Acta 659, 258–265. (10.1016/j.aca.2009.11.035) 20103133

[30] Essendoubi M, Toubas D, Bouzaggou M, Pinon J-M, Manfait M, Sockalingum GD 2005 Rapid identification of *Candida* species by FT-IR microspectroscopy. Biochim. Biophys. Acta 1724, 239–247. (10.1016/j.bbagen.2005.04.019) 15951116

[31] Corte L, Antonielli L, Roscini L, Fatichenti F, Cardinali G 2011 Influence of cell parameters in Fourier transform infrared spectroscopy analysis of whole yeast cells. Analyst 136, 2339–2349. (10.1039/c0an00515k) 21494743

[32] Cardinali G, Rellini P, Pelliccia C, Antonielli L, Fatichenti F 2008 MMS: a ‘R’ package for metabolomic markers search in stress response studies. Open Appl. Inform. J. 2, 1–8. (10.2174/1874136300802010001)

[33] Favaro L et al. 2016 A novel FTIR-based approach to evaluate the interactions between lignocellulosic inhibitory compounds and their effect on yeast metabolism. RSC Adv. 6, 47 981–47 989. (10.1039/C6RA08859G)

[34] Corte L, Tiecco M, Roscini L, De Vincenzi S, Colabella C, Germani R, Tascini C, Cardinali G 2015 FTIR metabolomic fingerprint reveals different modes of action exerted by structural variants of n-alkyltropinium bromide surfactants on *Escherichia coli* and *Listeria innocua* cells. PLoS ONE 10, e0115275 (10.1371/journal.pone.0115275) 25588017 PMC4294686

[35] Fuess LT, Garcia ML 2014 Implications of stillage land disposal: a critical review on the impacts of fertigation. J. Environ. Manage. 145, 210–229. (10.1016/j.jenvman.2014.07.003) 25058869

