# Supplementary material for: A yeast metabolome-based model for an ecotoxicological approach in the management of lignocellulosic ethanol stillage
Source: R Soc Open Sci. 2019 Jan 16;6(1):180718. doi: 10.1098/rsos.180718 (PMC6366221; doi:10.1098/rsos.180718)
Supplement: Supplementary Figures [file rsos180718supp1.pptx]

## Slide 1
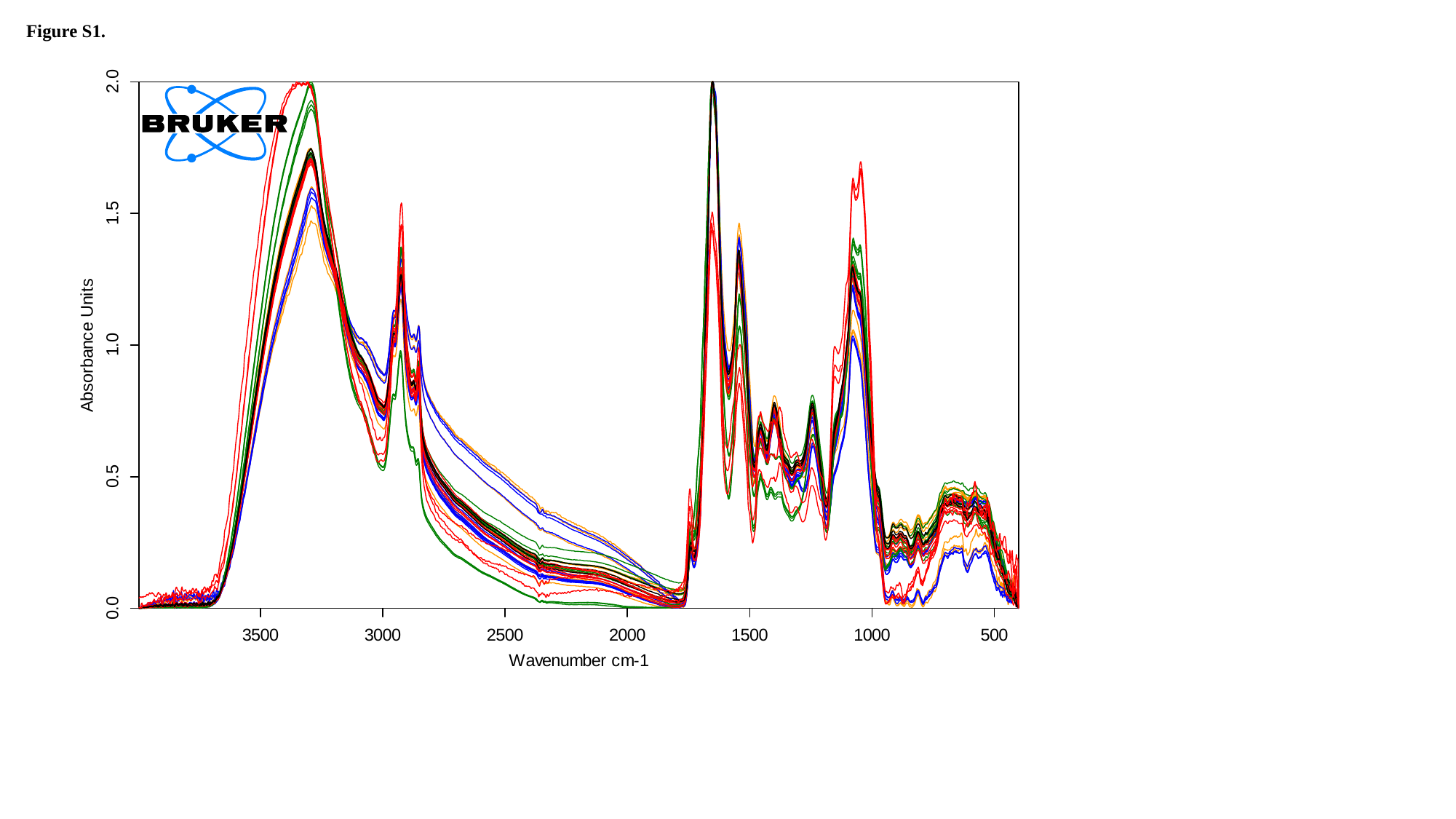

Figure S1.

## Slide 2
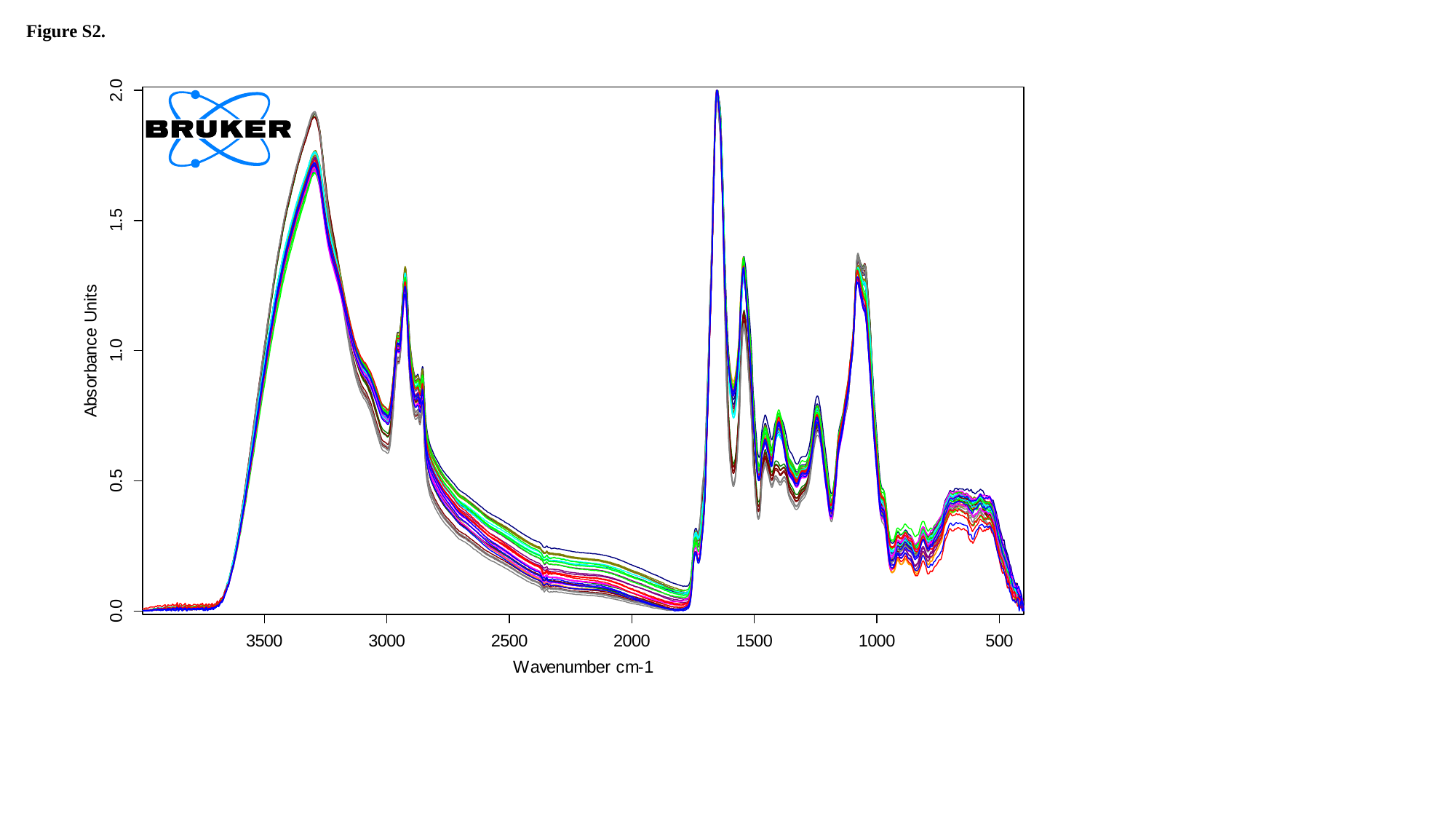

Figure S2.

## Slide 3
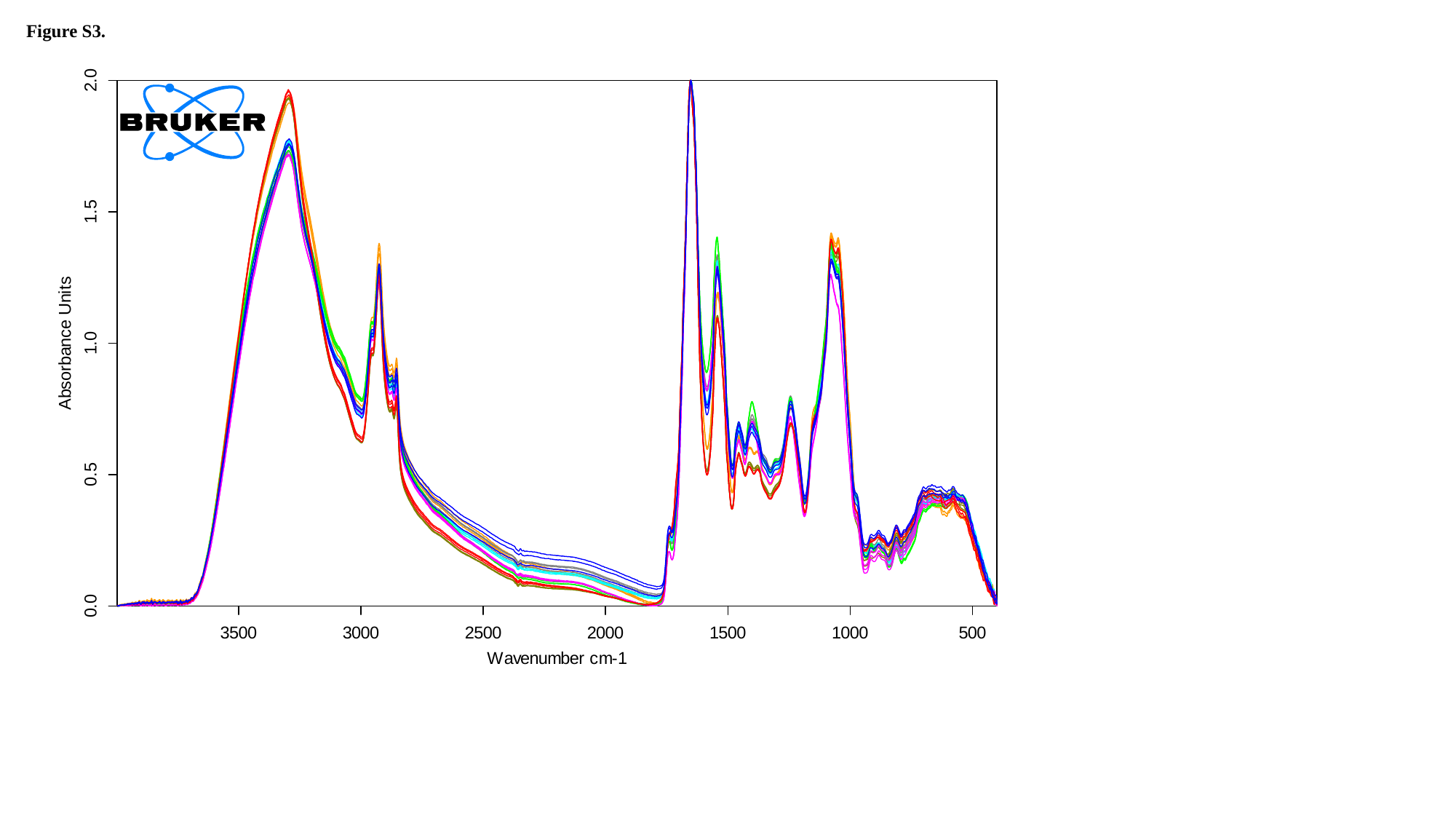

Figure S3.

## Slide 4
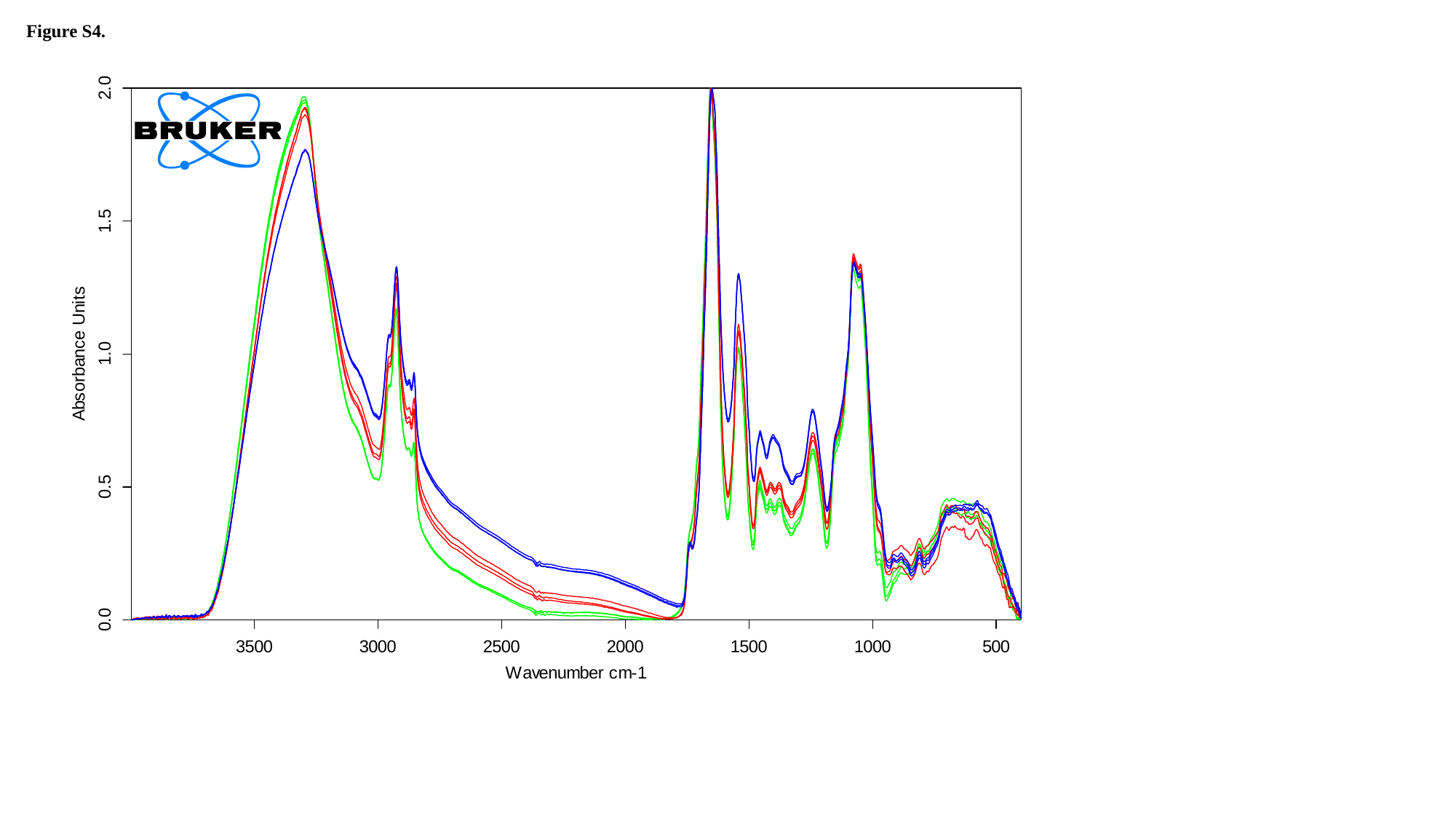

Figure S4.

## Slide 5
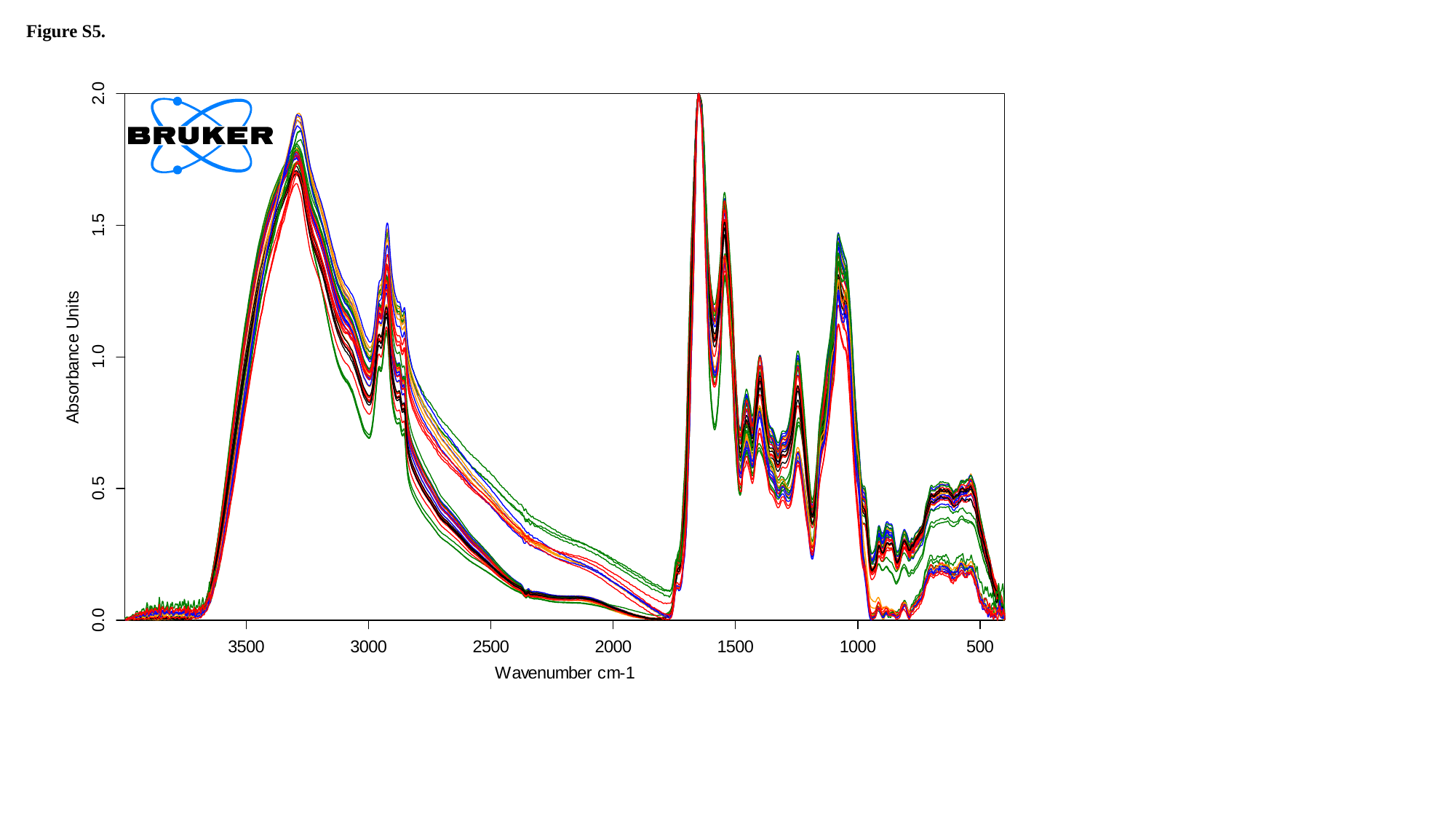

Figure S5.

## Slide 6
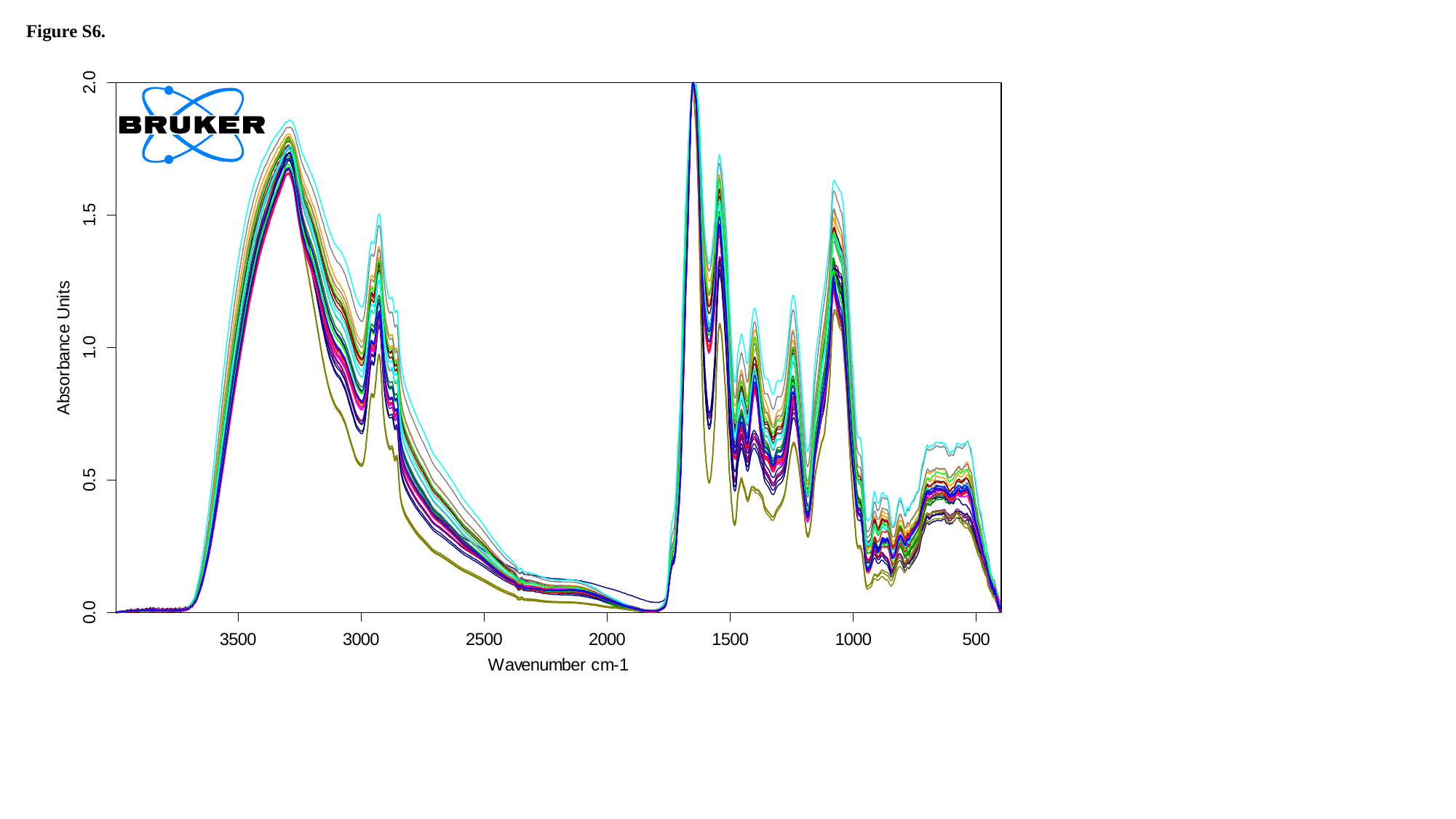

Figure S6.

## Slide 7
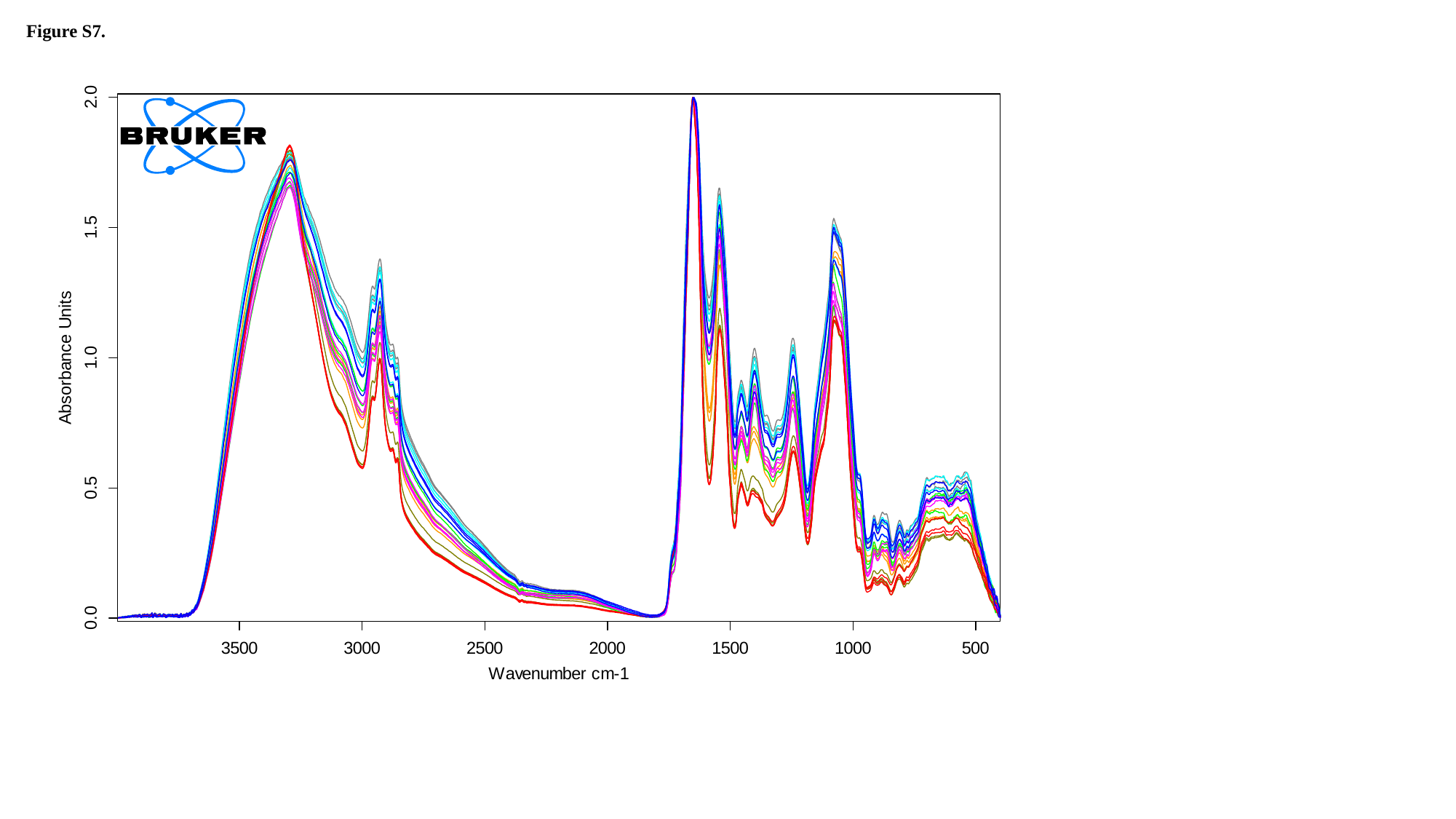

Figure S7.

## Slide 8
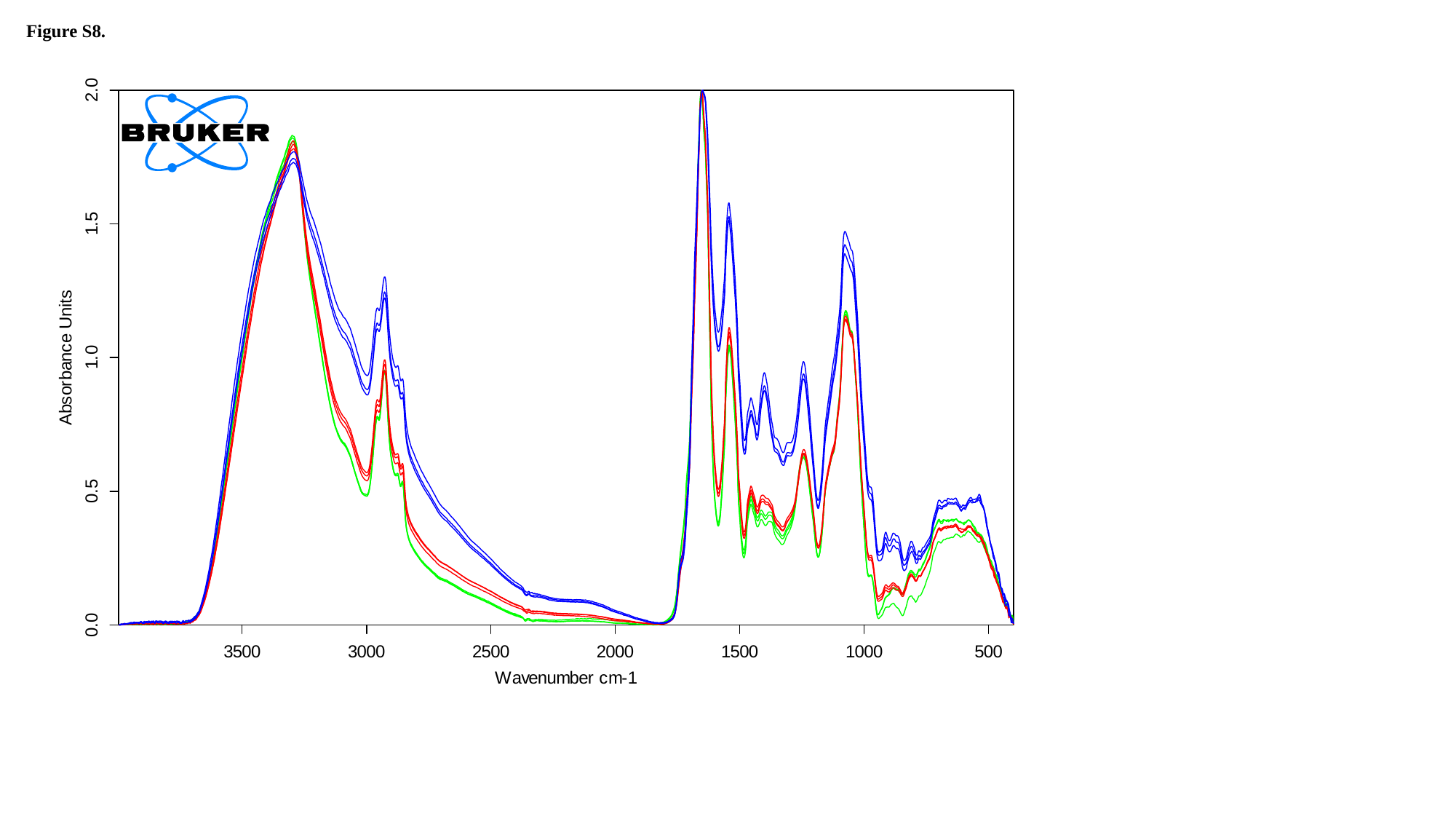

Figure S8.

## Slide 9
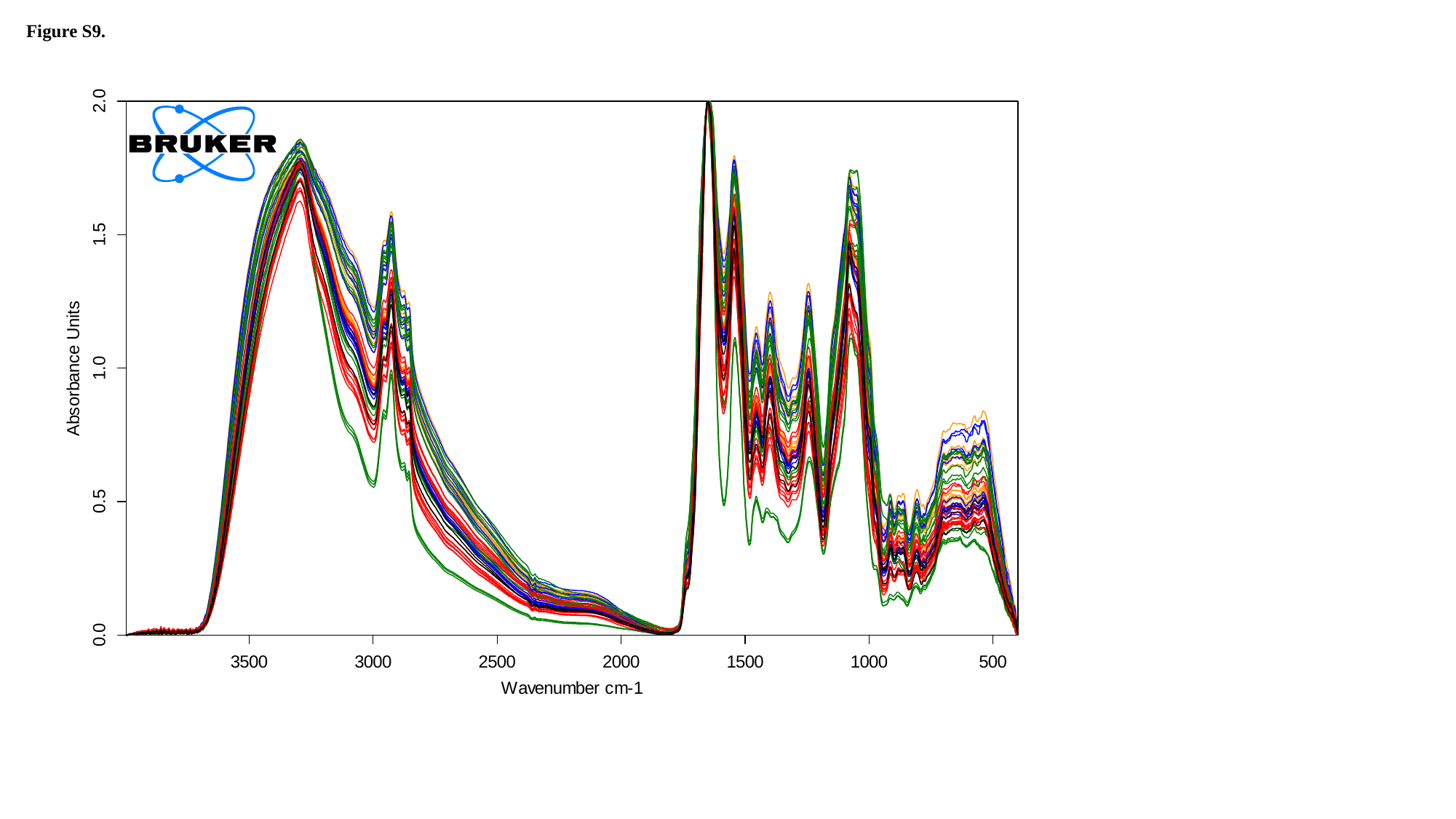

Figure S9.

## Slide 10
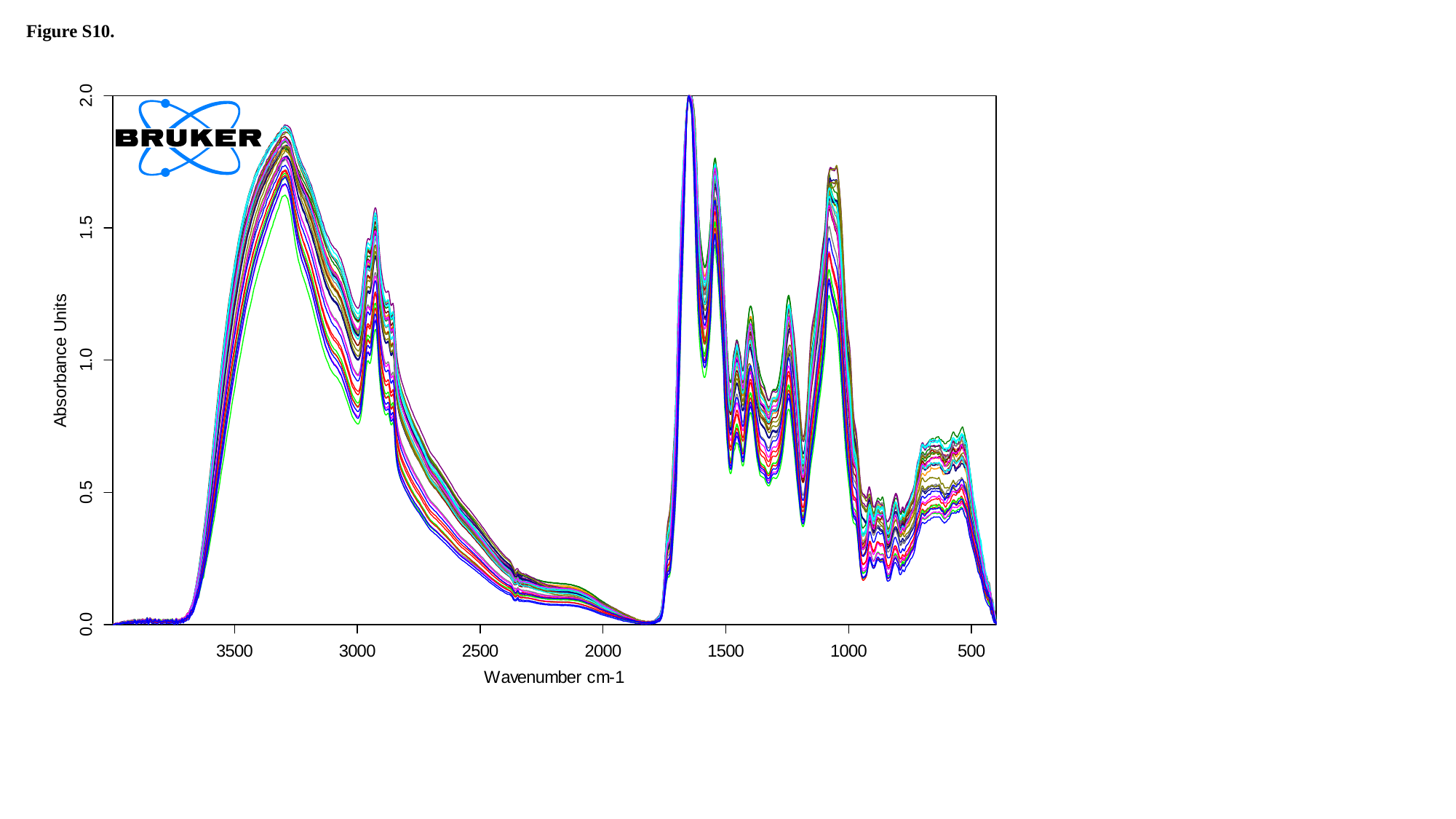

Figure S10.

## Slide 11
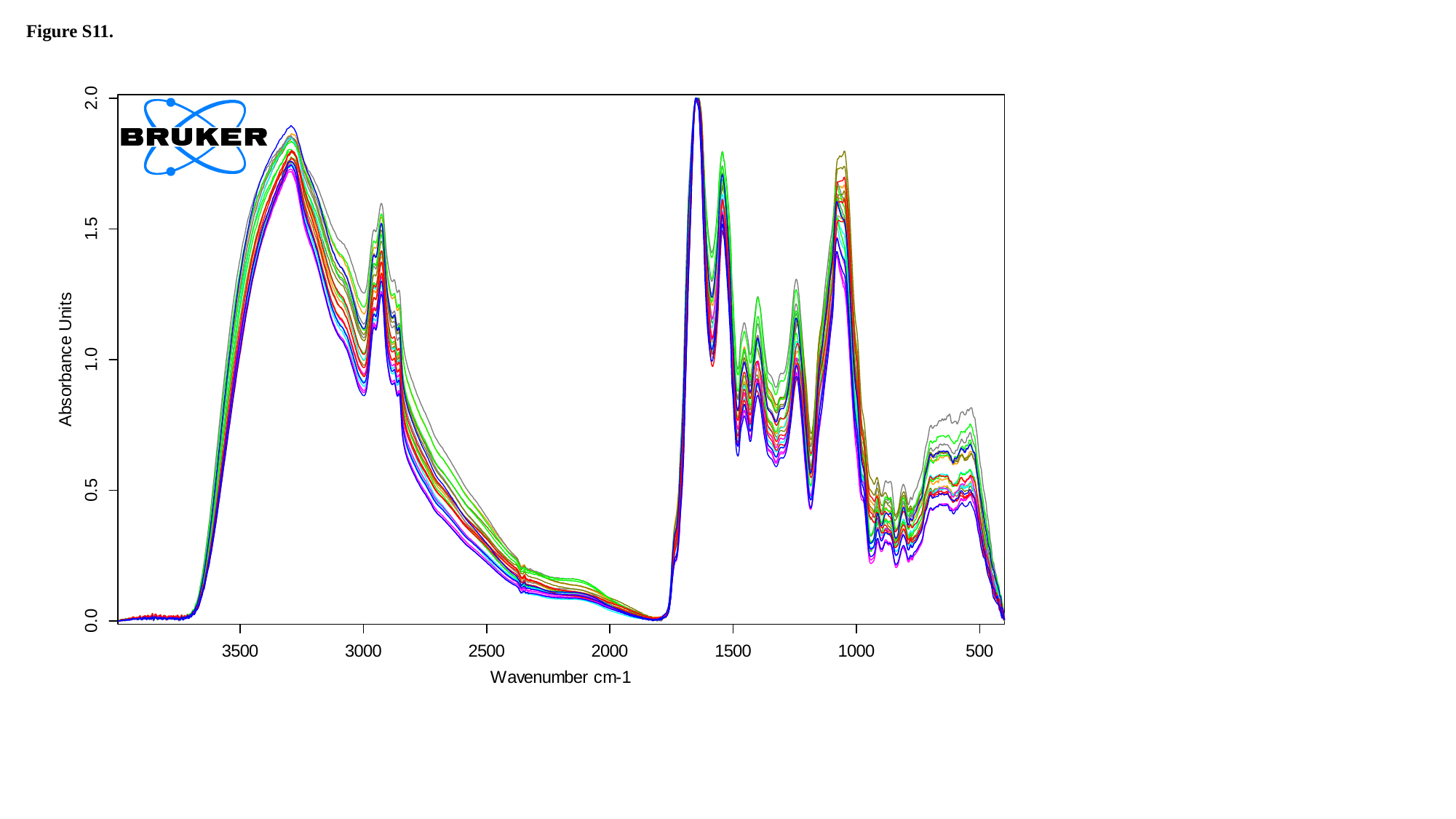

Figure S11.

## Slide 12
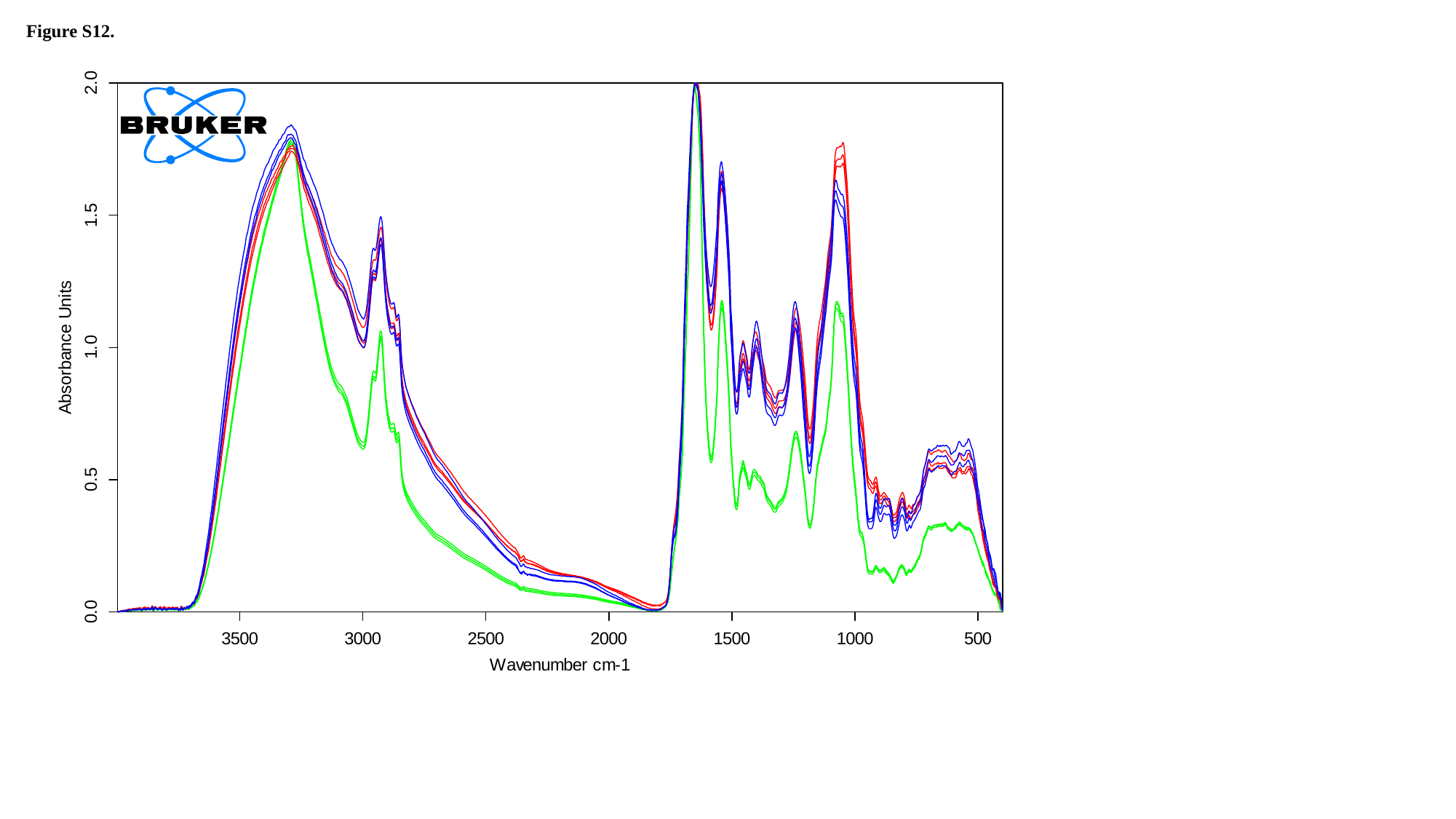

Figure S12.
