# Supplementary material for: A yeast metabolome-based model for an ecotoxicological approach in the management of lignocellulosic ethanol stillage
Source: R Soc Open Sci. 2019 Jan 16;6(1):180718. doi: 10.1098/rsos.180718 (PMC6366221; doi:10.1098/rsos.180718)
Supplement: Titles and captions of ESM [file rsos180718supp2.docx]

**Roscini et al-ESM**

**Title and captions of ESM**

**Figure S1.** FTIR spectra of DSM70449 cells exposed to the four inhibitors.

**Legend.** Each sample is presented in triplicate. The four compounds are represented by the different colors as detailed: Black = Control Sample; Red = Acetic Acid; Green = Formic Acid; Blue = Furfural; Orange = HMF.

**Figure S2.** FTIR spectra of DSM70449 cells exposed to the four inhibitors in binary mixtures.

**Legend.** Each sample is presented in triplicate. The binary mixtures are represented by the different colors as detailed: Blue = Acetic Acid RC_25_ + Furfural RC_25_; Red = Acetic Acid RC_50_ + Furfural RC_50_; Pink = Acetic Acid RC_25_ + HMF RC_25_; Light Green = Acetic Acid RC_50_ + HMF RC_50_; Light Blue = Acetic Acid RC_25_ + Formic Acid RC_25_; Dark Green = Acetic Acid RC_50_ + Formic Acid RC_50_; Grey = Formic Acid RC_25_ + Furfural RC_25_; Dark Blue = Formic Acid RC_50_ + Furfural RC_50_; Dark Red = Formic Acid RC_25_ + HMF RC_25_; Orange = Formic Acid RC_50_ + HMF RC_50_;

**Figure S3.** FTIR spectra of DSM70449 cells exposed to the four inhibitors in ternary mixtures.

**Legend.** Each sample is presented in triplicate. The ternary mixtures are represented by the different colors as detailed: Blue = Acetic Acid RC_25_ + Furfural RC_25_+ Formic Acid RC_25_; Red = Acetic Acid RC_50_ + Furfural RC_50_+ Formic Acid RC_50_; Pink = Acetic Acid RC_25_ + HMF RC_25_+ Furfural RC_25_; Light Green = Acetic Acid RC_50_ + HMF RC_50_+ Furfural RC_50_; Light Blue = Acetic Acid RC_25_ + Formic Acid RC_25_+ HMF RC_25_; Dark Green = Acetic Acid RC_50_ + Formic Acid RC_50_+ HMF RC_50_; Grey = Formic Acid RC_25_ + Furfural RC_25_+ HMF RC_25_; Orange = Formic Acid RC_50_ + Furfural RC_50_ + HMF RC_50_.

**Figure S4.** FTIR spectra of DSM70449 cells exposed to the four inhibitors in quaternary mixtures.

**Legend.** Each sample is presented in triplicate. The quaternary mixtures are represented by the different colors as detailed: Blue = Acetic Acid RC_25_ + Furfural RC_25_+ Formic Acid RC_25_+ HMF RC_25_; Red = Acetic Acid RC_50_ + Furfural RC_50_+ Formic Acid RC_50_+ HMF RC_50_; Light Green = Acetic Acid RC_100_ + HMF RC_100_+ Furfural RC_100_;+ HMF RC_100_.

**Figure S5.** FTIR spectra of Fm17 cells exposed to the four inhibitors.

**Legend.** Each sample is presented in triplicate. The four compounds are represented by the different colors as detailed: Black = Control Sample; Red = acetic acid; Green = formic acid; Blue = furfural; Orange = HMF.

**Figure S6.** FTIR spectra of Fm17 cells exposed to the four inhibitors in binary combinations.

**Legend.** Each sample is presented in triplicate. The binary mixtures are represented by the different colors as detailed: Blue = Acetic Acid RC_25_ + Furfural RC_25_; Red = Acetic Acid RC_50_ + Furfural RC_50_; Pink = Acetic Acid RC_25_ + HMF RC_25_; Light Green = Acetic Acid RC_50_ + HMF RC_50_; Light Blue = Acetic Acid RC_25_ + Formic Acid RC_25_; Dark Green = Acetic Acid RC_50_ + Formic Acid RC_50_; Grey = Formic Acid RC_25_ + Furfural RC_25_; Dark Blue = Formic Acid RC_50_ + Furfural RC_50_; Dark Red = Formic Acid RC_25_ + HMF RC_25_; Orange = Formic Acid RC_50_ + HMF RC_50_;

**Figure S7.** FTIR spectra of Fm17 cells exposed to the four inhibitors in ternary mixtures.

**Legend.** Each sample is presented in triplicate. The ternary mixtures are represented by the different colors as detailed: Blue = Acetic Acid RC_25_ + Furfural RC_25_+ Formic Acid RC_25_; Red = Acetic Acid RC_50_ + Furfural RC_50_+ Formic Acid RC_50_; Pink = Acetic Acid RC_25_ + HMF RC_25_+ Furfural RC_25_; Light Green = Acetic Acid RC_50_ + HMF RC_50_+ Furfural RC_50_; Light Blue = Acetic Acid RC_25_ + Formic Acid RC_25_+ HMF RC_25_; Dark Green = Acetic Acid RC_50_ + Formic Acid RC_50_+ HMF RC_50_; Grey = Formic Acid RC_25_ + Furfural RC_25_+ HMF RC_25_; Orange = Formic Acid RC_50_ + Furfural RC_50_ + HMF RC_50_.

**Figure S8.** FTIR spectra of Fm17 cells exposed to the four inhibitors in quaternary mixtures.

**Legend.** Each sample is presented in triplicate. The quaternary mixtures are represented by the different colors as detailed: Blue = Acetic Acid RC_25_ + Furfural RC_25_+ Formic Acid RC_25_+ HMF RC_25_; Red = Acetic Acid RC_50_ + Furfural RC_50_+ Formic Acid RC_50_+ HMF RC_50_; Light Green = Acetic Acid RC_100_ + HMF RC_100_+ Furfural RC_100_;+ HMF RC_100_.

**Figure S9.** FTIR spectra of Fp84 cells exposed to the four inhibitors.

**Legend.** Each sample is presented in triplicate. The four compounds are represented by the different colors as detailed: Black = Control Sample; Red = acetic acid; Green = formic acid; Blue = furfural; Orange = HMF.

**Figure S10.** FTIR spectra of Fp84 cells exposed to the four inhibitors in binary mixtures.

**Legend.** Each sample is presented in triplicate. The binary mixtures are represented by the different colors as detailed: Blue = Acetic Acid RC_25_ + Furfural RC_25_; Red = Acetic Acid RC_50_ + Furfural RC_50_; Pink = Acetic Acid RC_25_ + HMF RC_25_; Light Green = Acetic Acid RC_50_ + HMF RC_50_; Light Blue = Acetic Acid RC_25_ + Formic Acid RC_25_; Dark Green = Acetic Acid RC_50_ + Formic Acid RC_50_; Grey = Formic Acid RC_25_ + Furfural RC_25_; Dark Blue = Formic Acid RC_50_ + Furfural RC_50_; Dark Red = Formic Acid RC_25_ + HMF RC_25_; Orange = Formic Acid RC_50_ + HMF RC_50_;

**Figure S11.** FTIR spectra of strain Fp84 cells exposed to the four inhibitors in ternary mixtures.

**Legend.** Each sample is presented in triplicate. The ternary mixtures are represented by the different colors as detailed: Blue = Acetic Acid RC_25_ + Furfural RC_25_+ Formic Acid RC_25_; Red = Acetic Acid RC_50_ + Furfural RC_50_+ Formic Acid RC_50_; Pink = Acetic Acid RC_25_ + HMF RC_25_+ Furfural RC_25_; Light Green = Acetic Acid RC_50_ + HMF RC_50_+ Furfural RC_50_; Light Blue = Acetic Acid RC_25_ + Formic Acid RC_25_+ HMF RC_25_; Dark Green = Acetic Acid RC_50_ + Formic Acid RC_50_+ HMF RC_50_; Grey = Formic Acid RC_25_ + Furfural RC_25_+ HMF RC_25_; Orange = Formic Acid RC_50_ + Furfural RC_50_ + HMF RC_50_.

**Figure S12.** FTIR spectra of Fp84 cells exposed to the four inhibitors in quaternary mixtures.

**Legend.** Each sample is presented in triplicate. The quaternary mixtures are represented by the different colors as detailed: Blue = Acetic Acid RC_25_ + Furfural RC_25_+ Formic Acid RC_25_+ HMF RC_25_; Red = Acetic Acid RC_50_ + Furfural RC_50_+ Formic Acid RC_50_+ HMF RC_50_; Light Green = Acetic Acid RC_100_ + HMF RC_100_+ Furfural RC_100_;+ HMF RC_100_.
